# Analysis of Insulin in Human Breast Milk in Mothers with Type 1 and Type 2 Diabetes Mellitus

**DOI:** 10.1155/2012/296368

**Published:** 2012-03-05

**Authors:** T. J. Whitmore, N. J. Trengove, D. F. Graham, P. E. Hartmann

**Affiliations:** ^1^Biochemistry and Molecular Biology, School of Biomedical and Chemical Sciences, Faculty of Life and Physical Sciences, The University of Western Australia, UWA M310, 35 Stirling Highway, Crawley, WA 6009, Australia; ^2^Clinical Services, Royal Perth Hospital, GPO Box X2213, Perth, WA 6001, Australia; ^3^Obstetrics and Gynaecology, School of Women's and Infant's Health, The University of Western Australia, King Edward Memorial Hospital, Subiaco, WA 6008, Australia

## Abstract

Despite the important role that insulin plays in the human body, very little is known about its presence in human milk. Levels rapidly decrease during the first few days of lactation and then, unlike other serum proteins of similar size, achieve comparable levels to those in serum. Despite this, current guides for medical treatment suggest that insulin does not pass into milk, raising the question of where the insulin in milk originates. Five mothers without diabetes, 4 mothers with type 1, and 5 mothers with type 2 diabetes collected milk samples over a 24-hour period. Samples were analysed for total and endogenous insulin content and for c-peptide content. All of the insulin present in the milk of type 1 mothers was artificial, and c-peptide levels were 100x lower than in serum. This demonstrates that insulin is transported into human milk at comparable concentration to serum, suggesting an active transport mechanism. The role of insulin in milk is yet to be determined; however, there are a number of potential implications for the infant of the presence of artificial insulins in milk.

## 1. Introduction

Insulin, a key hormone in blood glucose homeostasis, has been detected in human colostrum at supraphysiological levels (114–306 mU/L) before decreasing to similar concentrations to those found in blood in the fasting state by day 5 postpartum in healthy mothers [1, 2]. Recent work by Tiittanen et al. [3] has demonstrated that insulin is also present in the milk of mothers with type 1 diabetes mellitus, who lack any endogenous insulin secretion from the pancreas. They also demonstrated that levels of insulin are significantly higher (*P* < 0.001) in milk from type 1 diabetic mothers than that of nondiabetic control mothers, at both days 3 to 7 postpartum and 3 months postpartum. Interestingly, insulin levels in the milk of control mothers vary greatly between studies, while those reported by Shehadeh et al. [4] showed higher milk insulin concentrations than those reported by Tiittanen et al. [3], suggesting that significant variation exists between mothers regardless of the presence of diabetes mellitus. However, the work by Tiittanen et al. [3] and Shehadeh et al. [4] does not further the time of collection or fasting state of mothers, which may account for some of the variability observed. In addition, Kulski and Hartmann [2] have demonstrated that the levels of insulin in breastmilk may vary with the stage of lactation, with high levels present in colostrum and levels decreasing to a plateau in established lactation. Despite this, current medical guides state that insulin is a peptide that is too large to be secreted into milk [5–7].

Given the established role of dietary insulin in the maturation of intestinal epithelium in rats, it can be postulated that insulin present in milk may play a similar role in the development of human intestinal epithelium [8, 9]. Additionally, work in rats has shown that a trypsin inhibitor present in milk acts to preserve insulin function [10, 11]. Furthermore, epithelial cell insulin receptors exist in the intestine of both piglets and calves, and insulin has been suggested to play a role in influencing growth and development of the small intestine [12, 13]. This would suggest that insulin could retain biological activity when ingested by the infant and that intact insulin may pass over from the gastrointestinal tract of infants into blood. This suggestion is further supported and extensively discussed in a review of the in vivo and in vitro effects of insulin on the gastrointestinal tract by Shehadeh et al. [14].

Endogenous insulin typically exists in blood in equilibrium between a free monomeric state and a hexamer with zinc; it is only able to bind to receptors in the free state and has a half-life of ~20 minutes [15]. A recombinant human protein is typically used for therapy. Due to the relatively short half-life, a number of short- or long-acting variants have been developed, either by modifying the amino acid sequence to prevent hexamer formation, or through complexing to zinc or protophane to shift the equilibrium towards the hexameric state [6, 16]. Due to these modifications to the amino acid sequence, specific assays can be used to identify whether the insulin present in a fluid sample is endogenous or a modified exogenous form of insulin [17, 18].

Tiitannen et al. [3] demonstrated that human dietary insulin in milk may have a tolerogenic effect against bovine insulin in formula and downregulate the production of IgG antibodies to bovine insulin, protecting against the development of autoimmunity, as shown with decreased levels of IgG to insulin in breast-fed children compared to those fed with cow's milk formula containing bovine insulin. Despite this beneficial effect at lower milk insulin levels, they also noted that high endogenous insulin levels in human milk were associated with a significantly increased incidence of *β*-cell autoimmunity in children, in both control mothers (*P* = 0.030) and mothers with type 1 diabetes (*P* = 0.045), which may suggest that exposure to high levels of endogenous insulin can also promote autoimmunity. However, it must be noted that this does not imply that development of type 1 diabetes mellitus is similarly promoted. In contrast to this, other recent work has demonstrated that administration of oral insulin may help protect residual *β*-cell function in children and adults with recent-onset type 1 diabetes [4, 19, 20]. Other work has supported the possible role of breast milk in the prevention of type 1 diabetes mellitus [21], since there is a lower incidence of type 1 diabetes in breast-fed children, but this is still a controversial area and contended by other sources [22–24]. The effects that type 2 and gestational diabetes mellitus may have on insulin concentration in milk have not yet been identified.

Given that the only known source of insulin in mothers with type 1 diabetes is injected exogenous insulin, this allows the hypothesis that either insulin is being transported into human milk, in contrast to current literature, or that it is being synthesised in the mammary gland and then secreted into the milk. There are currently no studies comparing the levels of exogenous insulin in milk compared to that produced endogenously in mothers with or without diabetes mellitus. The purpose of this study was to identify the origin of the insulin present in the milk of mothers with type 1 diabetes mellitus as either endogenous or exogenous by determining the levels of total insulin, endogenous insulin, and c-peptide in the milk compared to mothers without diabetes mellitus. This study also aimed to compare the levels of insulin in breast milk from mothers with type 2 diabetes and then investigate any relationship between blood glucose, milk insulin, glucose, c-peptide, and sodium content in breast milk in each of the groups. Mothers with type 2 diabetes mellitus have been included in order to compare insulin concentrations and as they should have no exogenous insulin present in their milk or blood. 

## 2. Methods

### 2.1. Patients

Samples from five control mothers (without diabetes mellitus) all in established lactation between 1 to 6 months were randomly selected from 24-hour production milk stock collected by the Lactation Research Group. Exact state of lactation is not further specified, as it was not relevant to the aims of this study in identifying the origin of insulin in milk. These mothers were designated T0-01 through T0-05. Mothers with diabetes mellitus were recruited from the Diabetes clinic at King Edward Memorial Hospital, Western Australia. Four mothers with type 1 diabetes mellitus were recruited, and all mothers in this group were on insulin replacement therapy. These mothers were designated T1-01 through T1-04. Five mothers with type 2 diabetes mellitus were recruited, and all mothers in this group was on dietary, exercise or metformin treatment, or a combination of these. None of the mothers in this group were on insulin replacement therapy. These mothers were designated T2-01 through T2-05. One mother in the type 1 group (T1-04) was lost to followup. All mothers in the three groups were breast feeding at the time and within 4 months postpartum. Details and demographic data for the mothers recruited are provided in Table 1. All mothers supplied written informed consent to participate, and the study was approved by the Human Research Ethics Committee, King Edward Memorial Hospital.

### 2.2. Milk Collection

Mothers were asked to perform a single 24-hour milk collection. This involved collecting 1 to 2 mL of fore and hind milk from each breast used at each feed for a period of 24 hours. Mothers fed infants 7–12 times per day from either one or both breasts. Fore and hind milk samples were collected from each feed. Feed times were at 2–4 hour intervals at all times of the day. There were a varying number of feeds per day per mother, depending on infant hunger and the breast used (single versus both). Samples were collected before and after a feed from each breast used for each feed. Some time points were a feed from a single breast—so samples were collected before and after the feed for that breast only. Other time-points involved a breastfeed from each breast so samples were collected before and after a feed from both the left and right breast. Sample analysis in this and previous studies has shown no difference in milk composition between left and right breasts so this data was analysed collectively. All samples were frozen by the mothers, and then transported to the laboratory and stored at −30°C prior for analysis. Samples were then thawed to 37°C, mixed, and centrifuged at 3,000 RPM (05PR-22 Refrigerated Centrifuge, Hitachi) for 10 minutes at 4°C in 300 *μ*L polypropylene tubes to separate the fat from the skim layer. Defatted milk was either immediately used for analysis or stored in appropriately labeled Eppendorf tubes (0.5 to 1.5 mL) and frozen at −30°C for later analysis.

### 2.3. Sample Analysis

Milk samples were analysed for glucose content using the spectrophotometric method described by Arthur et al. [25]. Defatted milk samples were analysed for total insulin content (endogenous + exogenous) using a chemiluminescent microparticle immunoassay (CMIA), which was performed at PathWest (PathWest Laboratory Medicine WA, QEII Medical Complex, Sir Charles Gairdner Hospital, Western Australia). Endogenous insulin content of defatted milk samples was measured using the Mercodia AB “Insulin ELISA” kit, as described in the kit insert [18]. C-peptide content of milk samples was similarly determined using the Mercodia AB “C-peptide ELISA” kit [26]. Total insulin content was additionally calculated using the Mercodia AB “Iso-Insulin ELISA” kit, along with the CMIA method described earlier [18]. The isoinsulin assay detects endogenous insulin, along with the exogenous insulin formulations (insulin aspart, detemir, glargine, glulisine, and lispro), compared to the standard insulin assay, which is specific for endogenous insulin only. Exogenous insulin content was calculated by subtracting endogenous insulin content from total (endogenous and all modified forms) insulin content [27]. Two techniques were used to analyse total insulin content (CMIA + ELISA) in order to assess suitability of the CMIA method for milk analysis; if appropriate, it could then be used in a larger-scale study as a more cost + time-effective approach to analysis of milk insulin content. The recovery, detection, and interassay coefficient of variation are presented in Table 2. Insulin content was measured for all samples, including fore + hind milk for each feed (*n* = 199).

Total fat content of the milk samples was assessed using the creamatocrit method described by Lucas et al. [28]. Finally, milk sodium analysis was performed using an atomic absorption spectroscopy approach, using a Varian Techtron absorption spectrophotometer model A100. Haemoglobin A_1c_ (HbA_1c_) was also documented from routine analysis in pregnancy for those mothers with diabetes.

### 2.4. Statistical Analysis

Although only a small number of mothers were present in each group, the large number of milk samples collected by each mother over the 24 hours periods allows for statistical analysis of differences between groups.

Differences between types of diabetes and between mothers were calculated using a repeated measures linear mixed-effects model, in order to take into account different numbers of feeds per day and differing numbers of mothers in each group. This was performed using the statistical functions of R (version 2.5.1, the R foundation for statistical computing). Unless otherwise stated, all results are presented as mean ± SEM. *P* values < 0.05 are considered significant.

## 3. Results

### 3.1. Insulin Content of Fore and Hind Milk

**Figure 1:**
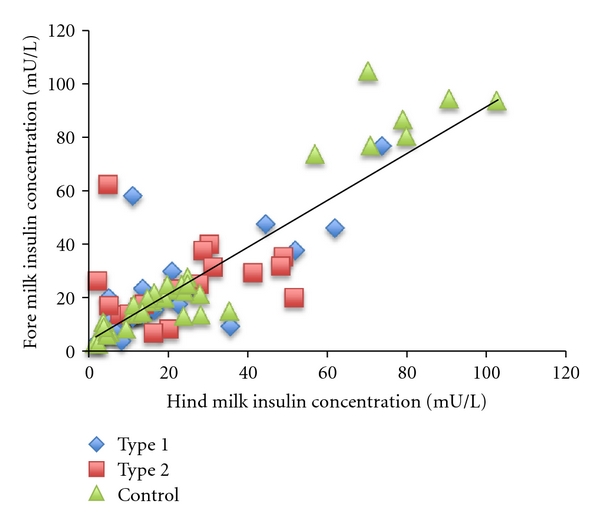
Correlation between total insulin content of fore and hind milk by CMIA method for all samples (*r* = 0.8413, *P* < 0.001, *n* = 199 all fore + hind milk samples).

Total insulin content of fore and hind milk samples was measured in all samples (*n* = 199) using the CMIA method and showed good correlation between the content of fore and hind milk samples when all mothers were considered, as shown in Figure 1. Analysis of individual groups showed no significant difference in insulin content of fore and hind milk between groups (*P* = 0.20, *P* = 0.98, and *P* = 0.14 for control, type 1, and type 2, resp.). Based on this, fore and hind milk insulin levels were averaged for the purpose of investigating any circadian variation; and this analysis showed no significant variation in insulin levels during different time points during the day. While there was circadian variation evident for all mothers, there was no consistent trend evident when analyzing as a group (data not shown).

### 3.2. Origin of Insulin in Human Milk

In order to determine whether the insulin present in the milk of mothers with type 1 diabetes is synthesised in the mammary gland or is transported from blood, total and endogenous insulin levels were measured in all milk samples in this group (*n* = 53). Exogenous insulin content was calculated as total insulin minus endogenous insulin for each sample. Mean ± SEM exogenous insulin levels in the milk of mothers with type 1 diabetes were 12.22 ± 1.34 mU/L for mother T1-01, 31.54 ± 4.95 mU/L for mother T1-02, and 17.86 ± 4.34 mU/L for mother T1-03. There was no detectable endogenous insulin content in the milk of mothers with type 1 diabetes; therefore, all insulin detected in the milk of mothers with type 1 diabetes was of exogenous origin.

### 3.3. Total Insulin Content in Human Milk

**Figure 2:**
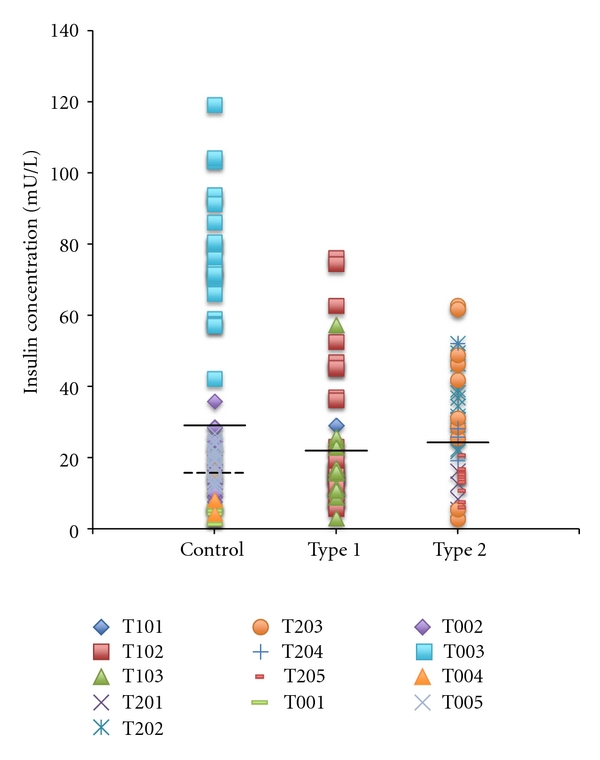
Total Insulin content of each milk sample (fore and hind milk) collected for all mothers as measured by the CMIA method. Dark lines indicate the mean for each group. The dotted line indicates the mean for the control group with the outlier mother T0-03 removed.

In order to investigate variations in insulin concentration between control mothers and mothers with diabetes, total insulin content of each milk sample (fore + hind milk individually) from each mother was measured using the CMIA technique (*n* = 199). There was no significant difference between fore and hind milk insulin content, so further data algorithmically treats each data point as the mean of fore + hind milk for each feed (time point). Total insulin content from all samples is presented in Figure 2, along with the mean insulin content of each group. The control mother T0-03 demonstrated significantly higher milk insulin content than the other control mothers (78.23 ± 3.96 versus 15.46 ± 1.03 mU/L, *P* < 0.001). There was no significant difference in mean insulin content between control mothers and mothers with type 1 diabetes (*P* = 0.74), control mothers and mothers with type 2 diabetes (*P* = 0.93), or between mothers with type 1 and type 2 diabetes (*P* = 0.62). 

### 3.4. C-Peptide Content of Human Milk

 Insulin is produced by cleaving insulin and c-peptide from the precursor molecule proinsulin. Hence, c-peptide content of the milk from two mothers with type 1 diabetes and 3 control mothers was measured using the ELISA method described (*n* = 61). C-peptide was detected in all samples; however, levels in the milk of control mothers were significantly higher than those in mothers with type 1 diabetes (21.09 ± 1.00 versus 14.49 ± 1.47 pmol/L, *P* < 0.01). There was no significant correlation between c-peptide and total insulin content in the milk of control mothers (*P* = 0.79) or mothers with type 1 diabetes (*P* = 0.85).

### 3.5. Sodium Content of Human Milk

**Figure 3:**
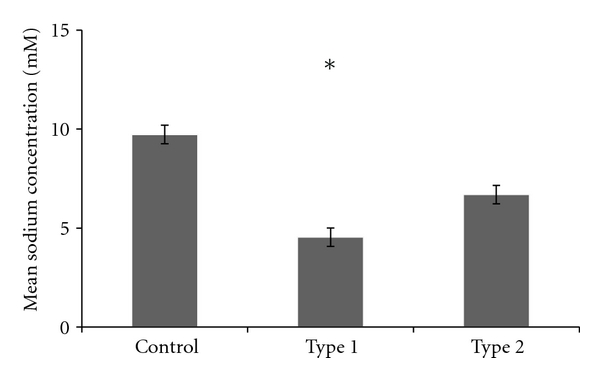
Mean sodium content of milk in each group. *indicates significant difference (*P* < 0.05) compared to control mothers.

In order to investigate the transport of insulin into human milk, the sodium content of the milk from all mothers was measured using atomic absorption spectroscopy (*n* = 199) as a marker of permeability of the paracellular pathway [29]. Mean sodium concentrations in milk are presented in Figure 3.

There was significantly lower sodium content in the milk of mothers with type 1 diabetes compared to control mothers (*P* < 0.05), but no significant difference in mean sodium content between control mothers and mothers with type 2 diabetes (*P* = 0.09) or between types of diabetes (*P* = 0.54). There was no significant relationship between sodium and insulin content of milk for control mothers (*P* = 0.17) or mothers with type 1 diabetes (*P* = 0.37), but a mild negative correlation existed for mothers with type 2 diabetes (*r* = −0.34, *P* < 0.05). Additionally, for the outlier control mother T0-03 who demonstrated significantly elevated insulin levels in milk, there was no significant relationship between sodium and insulin content in milk (*P* = 0.61).

### 3.6. Blood Glucose Levels and Milk Insulin Content

In order to investigate any correlation between blood glucose levels and insulin content in the milk of mothers with type 1 and 2 diabetes, mothers were asked to record their blood glucose levels at least 4 times over the course of the same 24-hour period as their milk sample collection. There was a great deal of variation within mothers for each group over a 24-hour period for both milk insulin and blood glucose; however, no significant consistent trends were identified. However, there was no significant difference (*P* = 0.15) in mean blood glucose levels between mothers with type 1 and type 2 diabetes. Mean blood glucose levels in mothers with type 1 diabetes were 8.52 ± 0.57 mmol/L, and levels for mothers with type 2 diabetes were 7.45 ± 0.26 mmol/L (mean ± SEM).

There was no significant relationship between mean blood glucose levels and mean milk insulin levels in mothers with type 1 diabetes (*P* = 0.72) or mothers with type 2 diabetes (*P* = 0.16). By comparing blood glucose levels and milk insulin concentration over time, there appears to be an inverse relationship between the two; a reduction in blood glucose levels is mirrored by an increase in milk insulin content. However, this does not hold true for all mothers. Mother T2-05 (type 2) demonstrated only small variability in milk insulin content, and mother T2-03 (type 2) demonstrated large variability in milk insulin content with only small variation in blood glucose levels. Limited blood glucose data was provided by mothers due to sample collection difficulties, and further comparison is not possible within the scope of this paper.

### 3.7. Glucose Content of Human Milk

**Figure 4:**
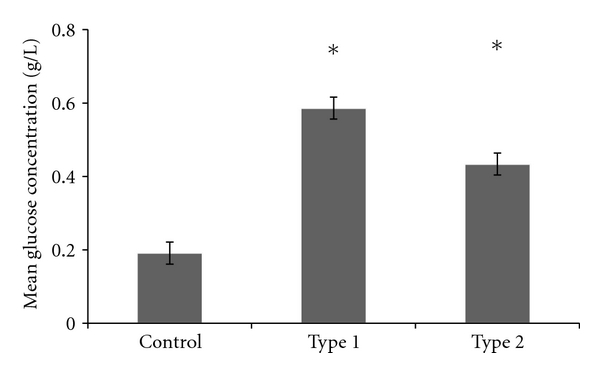
Mean glucose content of milk in each group. *indicates significant difference (*P* < 0.05) compared to control mothers.

As the glucose present in human milk derives from blood glucose, there may be fluctuations in milk glucose content that correlate with either blood glucose levels or insulin content of milk. Glucose content of each sample (fore and hind milk) was measured using the spectrophotometric method described. Mean glucose concentration in each group is shown in Figure 4.

There was significantly higher glucose content in the milk of mothers with type 1 diabetes compared to control mothers (*P* < 0.05) and in the milk of mothers with type 2 diabetes compared to control mothers (*P* < 0.05), but no significant difference in milk glucose content between types of diabetes (*P* = 0.68). There was no significant correlation between milk and blood glucose levels for mothers with type 1 diabetes (*P* = 0.60) or mothers with type 2 diabetes (*P* = 0.33). There was no significant relationship between glucose and insulin content of milk in control mothers (*P* = 0.17) or mothers with type 2 diabetes (*P* = 0.11), but there was a slight negative correlation between glucose and insulin content in milk from mothers with type 1 diabetes (*r* = −0.41, *P* < 0.01).

## 4. Discussion

Most current medical references state that insulin is too large to cross over from blood into milk [5–7]. However, there have been continuous references in the scientific literature to the presence of insulin in milk and the presence of elevated levels in the milk of mothers with diabetes compared to mothers without diabetes [1–3, 30]. Based on this conflicting evidence, it is proposed that insulin could either be transported into milk or be synthesised in the mammary gland and directly secreted into milk. This is a novel concept and first proposed in this pilot study.

### 4.1. Origins of Insulin in Human Milk

The origin of insulin in milk was determined through two approaches. Firstly, the type of insulin present in the milk of mothers with type 1 diabetes mellitus was assessed. These mothers should have no endogenous insulin production from the pancreas, and all insulin present in their blood should result from injections of exogenous insulin. Analysis of the milk of these mothers revealed that there was no endogenous insulin present, suggesting that it was all derived from injected insulin present in blood.

Secondly, the presence of c-peptide in the milk of two mothers with type 1 diabetes and three control mothers was measured. As the endogenous ELISA used is highly specific for human insulin, it was possible that any insulin synthesised in the mammary gland may be sufficiently structurally different to insulin synthesised in the pancreas as to not be detected by the ELISA, as is seen the two human isoforms of apolipoprotein B (ApoB48, synthesized in the small intestine, and ApoB100, synthesized in the liver) [31]. As insulin and c-peptide are cleaved from proinsulin in equimolar quantities [32], if insulin was synthesised in the mammary gland, then there should be equimolar quantities of both present in milk. C-peptide was identified in milk mothers with type 1 diabetes, but at 15 to 20x lower than reference blood concentrations [33], despite the common view that patients with type 1 diabetes do not produce insulin. However, many patients with type 1 diabetes still retain some residual pancreatic *β*-cell function, and insulin is secreted at subphysiological levels alongside c-peptide [34]. These factors indicate that the insulin present in milk is derived from insulin in blood.

### 4.2. Transport of Insulin into Human Milk

 Insulin content in milk for all groups was found to be similar to known reference levels for blood. This is the first time insulin levels have been analysed in milk from mothers with type 2 diabetes and is in concordance with previous work investigating insulin concentration in the milk of control mothers and mothers with type 1 diabetes [1–3]. In contrast to other serum proteins of similar size, which are normally present in milk at up to 100x lower concentration than found in blood [35], these results suggest that insulin does not diffuse into milk via the paracellular pathway. This is supported by the lack of any correlation between insulin and sodium content of milk for control mothers and mothers with type 1 diabetes; as sodium content of milk is an indicator of the permeability of the paracellular pathway, a positive correlation between insulin and sodium content of milk would be expected if insulin was entering via the paracellular pathway. Additionally, sodium content was significantly lower in mothers with type 1 diabetes than in controls, suggesting that the paracellular pathway is closed in these mothers. There was no significant difference in sodium content of milk between mothers with type 2 diabetes and control mothers.

These findings suggest that insulin must be actively transported into milk in order to achieve these higher concentrations. Two insulin transporters have currently been identified; an active transporter present on the blood brain barrier (BBB) and receptor-mediated endocytosis of insulin occurring in skeletal muscle endothelium [36, 37]. As the BBB transporter is only capable of establishing a cerebral concentration of less than 10% of the systemic blood concentration, it is unlikely to be involved in transport of insulin in the mammary gland. However, the transport system in the skeletal muscle endothelium is capable of transporting insulin at up to 50% of blood levels. While this is still lower than the ~100% transfer seen in the mammary gland, as milk is synthesised over a period of time, this could potentially account for the high concentrations present in milk. Further consideration must be given to the supraphysiological insulin concentrations demonstrated in human colostrum, and how these are achieved [2]. While this study did not assess colostrum insulin content, it can be postulated that the potential milk trypsin inhibitor postulated in rat models may act to preserve and stabilize insulin within milk, leading to these higher concentrations [10, 11]. Alternately, active transport of insulin into milk may increase in the colostrum phase, particularly if insulin does indeed play a large role in maturation of the infant digestive system [13, 14]. Further research into insulin transport mechanisms in both colostrum and normal milk is required to better investigate this possibility.

There was no significant difference between the levels of insulin in the milk of control mothers, mothers with type 1, and mothers with type 2 diabetes, despite the insulin present in the type 1 mothers being artificial in nature. This suggests that the exogenous insulin used for treatment of type 1 diabetes is transported into milk with similar affinity to endogenous insulin in mothers without diabetes.

As the exact transport mechanism has yet to be scientifically quantified, the significantly elevated milk insulin levels in control mother T0-03 cannot be readily explained. If milk insulin concentration does reflect or mirror blood insulin concentrations, then the elevated insulin levels seen would typically represent a woman with undiagnosed, untreated type 2 diabetes [38]. However, as the transporter has yet to be identified, the possibility remains that the elevated milk insulin levels in this mother are due to altered insulin transport, rather than elevated blood insulin levels; as two isoforms of the insulin receptor exist, it is possible that the high specificity isoform may be preferentially expressed in the mammary gland of this mother, thus resulting in increased insulin transport into her milk [39].

### 4.3. Blood Glucose Levels and Milk Insulin Concentration

 Varying data exists in the literature as to the glucose content of milk in mothers with diabetes. It has been established by Neville et al. [29] that glucose transport into milk is insulin independent and dependent upon blood glucose concentrations. Given this, it would be expected that patients with diabetes would have elevated milk glucose levels, reflecting their typically elevated blood glucose levels [15]. This study identified significantly higher milk glucose concentrations in mothers with type 1 and type 2 diabetes compared to control mothers, as was predicted. There was no correlation with maternal blood glucose levels, in contrast to data reported by Jackson et al. [40]. However, this may be a result of only being able to compare the mean blood and milk glucose levels for each mother; the study by Jackson et al. [40] compared the direct relationship between blood glucose and milk glucose at time of expression. This finding and the inverse relationship have been included as a potential important course of future investigation.

### 4.4. Potential Roles of Milk Insulin

 This study demonstrated that both exogenous and endogenous insulin, are actively transported into human milk in similar concentrations to those found in blood, in comparison to other serum proteins of similar or smaller size, such as c-peptide. Presumably milk insulin must therefore play a functional or developmental role in the infant. Studies in a rat model have demonstrated that oral insulin in milk is involved in maturation of intestinal epithelium and induces pancreatic amylase development at weaning [8, 10, 11]. It has also been demonstrated to influence lactase and saccharase activity in the small intestine in a piglet model [13]. A series of papers reported by Koldovský [1] show that human infants demonstrate decreased blood glucose levels in response to milk insulin in early development, suggesting that intact insulin is crossing into the bloodstream of the infant. Similarly, work by Mosinger et al. [41] also showed that suckling rats exhibit decreased blood glucose levels in response to oral insulin, whereas weaned rats do not. This is of particular importance in neonatal care, as infants of mothers with diabetes are frequently retained in the neonatal intensive care unit and fed expressed breast milk from their mothers [42]. These infants could potentially then remain hypoglycaemic for longer periods, in contrast to the intended aim of the protocol. 

Tiittanen et al. [3] showed that dietary nonhuman insulin can potentially act as an immunogen and predispose the infant to formation of anti-insulin antibodies, while low levels of human insulin in milk can act as a tolerogen to downregulate any immune response to foreign dietary insulin. Their study investigated the presence of bovine insulin in formula as the potential immunogen, which is only three amino acids different in sequence to human insulin. Given this potential immunogenic role of nonhuman insulin, it is important to consider the potential implications of the presence of artificial insulin in human milk. Artificial recombinant human insulins that are currently used as treatment for patients with type 1 diabetes are typically also only one to three amino acids different in sequence to human insulin [17]. It can therefore be hypothesised that the presence of artificial insulin in the milk of mothers with type 1 diabetes may be acting as an immunogen, which could potentially increase the risk of the infant developing auto-immune diseases in later life. As this immunogenic effect was demonstrated in both early and late lactation, it is possible that insulin is being degraded and only peptide fragments are entering the blood from the intestine. However, given the difference noted in immunogenicity, it is likely that those breakdown products that enter blood still contain the sequence regions that differ between recombinant and endogenous insulin. However, the data in the literature of the role of breastfeeding on the development of type 1 diabetes mellitus in control mothers and those with type 1 diabetes mellitus is still controversial and that the rate of diabetes mellitus in children of type 1 diabetic mothers is lower than in the general population, further suggesting a potential tolerogenic effect of milk insulin [22–24].

### 4.5. Limitations

A number of limitations must be taken into consideration when interpreting the results of the above study. Firstly, as a pilot study, there are only a small number of participants and hence a low sample size. A repeated measures model and a large number of samples per participant are used to counter this, but the low sample size may limit the interpretation of trends of other milk components. However, this should not affect interpretation of human milk insulin origin as exogenous or endogenous. Further, the specificity of the ELISA assays used for identification of human pancreatic insulin compared to modified exogenous insulin forms means that if there were any structural differences between human pancreatic and a hypothetical mammary source, then the mammary insulin may not be detected with this assay. Finally, there was a significant lack of blood glucose concentration data to compare to milk glucose and milk insulin, due to collection difficulties with mothers involved in this study.

### 4.6. Future Directions

 This study has identified that both endogenous and exogenous insulin is transported from blood into human milk. However, the exact method of transfer still needs to be identified and characterised. Studies in a rat model with labelled artificial insulin could potentially be used to identify the source of transfer into milk, and use of a hyperinsulinaemic clamp model may be used to determine (a) the rate of transfer and (b) the transport maximum for the mammary gland transporter.

Given that insulin is actively transported into human milk and is protected from degradation, it presumably plays a functional or developmental role in the infant. Given this, the presence of insulin in formula products needs to be reassessed; as not all mothers will breast-feed their infants, if insulin plays a functional or developmental role then the addition of insulin to formula could potentially act to improve the similarities of formula to human milk and thus be beneficial to the health and development of formula-fed infants.

## Figures and Tables

**Table 1:** Demographic details of mothers with diabetes.

ID	Age	Type of diabetes	HbA_1c_	Method of delivery^A^	Medications	Stage of lactation
T1-01	23	1	6.7	V	Novorapid + lantis	5 months
T1-02	36	1	5.4	C	Novorapid + lantis	4 months
T1-03	32	1	4.8	C	Novorapid + lantis	2.5 months
T1-04*	27	1	7.4	C (E)	Novorapid + lantis	2 months
T2-01	32	2	5.9	V	Metformin + diet	4 months
T2-02	32	2	5.1	V (A)	Metformin + diet	3 months
T2-03	37	2	—	C	Metformin + diet	N/A <6 months
T2-04	27	2	6.0	C	Diet	3 months
T2-05	27	2	5.0	V	Diet	10 days

^A^V denotes vaginal delivery; A denotes assisted; C denotes Caesarean section; E denotes emergency.

*mother lost to followup.

**Table 2:** Insulin assay verification.

Assay	Recovery	Detection limit	Interassay coefficient of variation
Insulin CMIA	100.91 ± 4.99%	1.32 mU/L	0.62%
Endogenous insulin ELISA	95.00 ± 3.60%	0.188 mU/L	2.69%
Total insulin ELISA	96.91 ± 7.69%	4.93 mU/L	9.49%
